# The Importance of Self-Determination to the Quality of Life of People with Intellectual Disability: A Perspective

**DOI:** 10.3390/ijerph17197121

**Published:** 2020-09-29

**Authors:** Michael L. Wehmeyer

**Affiliations:** Department of Special Education, School of Education and Human Sciences, Joseph R. Pearson Hall, University of Kansas, 1122 W. Campus Road, Room 521, Lawrence, KS 66045-3101, USA; wehmeyer@ku.edu; Tel.: +1-785-864-0723

**Keywords:** self-determination, quality of life, intellectual disability, causal agency, volitional action, agentic action, action-control beliefs, choice

## Abstract

There is both an intuitive and theoretical link between self-determination and quality of life for people with intellectual and developmental disabilities. Theoretically, definitions of self-determination have framed the construct with regard to its contribution to a person’s overall quality of life, while theoretical frameworks of quality of life have included self-determination among the core dimensions contributing to enhanced quality of life. These theoretical linkages have been supported by research on the quality of life and self-determination of people with intellectual and developmental disabilities and the relationships between these constructs. This article provides an overview of theoretical frameworks of self-determination, their relationship with theoretical frameworks of quality of life, and research pertaining to these constructs with people with intellectual and developmental disabilities. It is concluded that self-determination and quality of life are important constructs in designing supports that enable people with intellectual and developmental disabilities and that an important means to enhance the quality of life of people with intellectual and developmental disabilities is to promote and enable people to be self-determined.

## 1. Introduction

There has been building, over the past four decades, a movement toward the application of strengths-based approaches to supporting people with intellectual and developmental disabilities [1]. It can be argued that the earliest contributions to this movement came with the application of the quality of life construct to the field [2]. The quality-of-life construct has evolved from one that was tied only to subjective perceptions of people to one in which the field “had … embraced the QOL concept as a sensitizing notion and an overarching principle for service delivery” [3] (p. 3). Schalock and Keith defined quality of life as “a multidimensional phenomenon composed of core domains influenced by personal characteristics and environmental factors” [3] (p. 11). The quality-of-life conceptual model that has driven the application of this construct as an overarching principle for service delivery, as forwarded by Schalock and Keith, consists of eight domains, or core dimensions, namely personal development, self-determination, interpersonal relations, social inclusion, rights, emotional well-being, physical well-being, and material well-being [3,4].

However, another construct that has been instrumental in the shift from a deficits approach to intellectual and developmental disabilities to a strengths-based approach is the self-determination construct [5,6]. In fact, the first mention of the importance of self-determination to the lives of people with intellectual and developmental disabilities occurred in a 1972 chapter by Swedish philosopher Bengt Nirje, who argued for the right of people with intellectual disability to live ‘normalized’ lives and experience self-determination [7]. It was another 20 years, however, before a focus on self-determination and people with intellectual and developmental disabilities returned [5], and that focus emerged along with the recognition of the importance of the quality-of-life construct as an overarching principle for services and supports [6]. Too often over the years, however, the contribution of self-determination to quality of life has been taken as a given. The purpose of this Perspective is to provide a theoretical basis for understanding self-determination that has utility for, in turn, understanding it as a core dimension of quality of life and, further, in understanding the importance of self-determination as contributing to quality of life of people with intellectual disability in public health and human rights domains. The article begins with a brief introduction to the quality of life framework, followed by an overview of self-determination as it has been conceptualized within the field of intellectual disability. That, in turn, is followed by consideration of the research that has examined relationships between self-determination and quality of life. Finally, suggestions for future research are discussed.

## 2. Quality of Life

There is no intent to provide a comprehensive overview of quality of life in this article, in part because that will be evident to readers of this topical issue from other articles and because there is insufficient space to do this justice. In our work, we have adopted the quality-of-life framework forwarded by Robert Schalock [1]. Schalock and Verdugo [8] identified five overarching ways in which a focus on quality of life in the field of intellectual disability has, in fact, benefitted people with intellectual and developmental disabilities, and several of these speak to the ongoing importance of quality of life in moving toward strengths-based approaches to intellectual disability. Quality of life, argued Schalock and Verdugo, has provided a framework for a service delivery system that is “based on the values of dignity, equality, empowerment, self-determination, non-discrimination, and inclusion” [8] (p. 46). Quality of life is not a “thing” that people have; it is a multidimensional construct that provides a means to design and evaluate supports for people in service systems [9]. Research using this framework has identified eight core quality-of-life dimensions: emotional well-being, interpersonal relationships, material well-being, personal development, physical well-being, self-determination, social inclusion, and rights [10]. However, it is also worth noting that, in addition to being a core dimension of quality of life, self-determination with Schalock’s framework is also a value upon which services are based and, presumably, an outcome of such services and supports.

## 3. Self-Determination

The self-determination construct was first applied to the intellectual disability context in the early 1990s [11]. Through the 20th century, the construct had been applied within personality and motivational psychology and in the field of social work and welfare. The common themes in how the construct has been used across these disparate fields was that self-determination fundamentally refers to people acting as causal agents; that is, as acting volitionally to make or cause things to happen in one’s life [12]. Within the field of intellectual disability, Wehmeyer defined self-determination as “acting as the primary causal agent in one’s life and making choices and decisions regarding one’s quality of life free from undue external influence or interference [13] (p. 30). It is important to note that, even at the earliest stage of the application of the self-determination construct to the field of intellectual disability, it was, by definition, linked to quality of life.

The most recent iteration of the theoretical framework introduced by Wehmeyer is Causal Agency Theory [14]. Briefly, Causal Agency Theory was proposed to explain how people become self-determined and to explicate the development of self-determination [15]. Causal Agency Theory defined self-determination as follows:

… *a dispositional characteristic manifested as acting as the causal agent in one’s life. Self-determined people (i.e., causal agents) act in service to freely chosen goals. Self-determined actions function to enable a person to be the causal agent in his or her life.*

[14] (p. 258)

Acting as a causal agent implies that a person makes or causes things to happen in their life. Self-determined people act to accomplish specific ends or to cause or create change in their lives. Acting in a self-determined manner implies that people make or causes things to happen in their own lives, rather than someone or something else making them act in other ways. Self-determined action is goal-oriented, driven by preferences and interests, and ultimately serves to enable people to enhance the quality of their lives [16].

Causal Agency Theory is grounded in human agentic theories that assume that action is self-caused and that people want to be the origin of their own behavior [17]. Self-determined action refers to the degree to which action is volitional and agentic, driven by beliefs about the relationships between actions (or means) and ends. Causal Action Theory posits three essential characteristics of self-determined action—volitional action, agentic action, and action-control beliefs—that contribute to causal agency and the development of self-determination. These essential characteristics refer not to specific actions performed or the beliefs that drive action, but to the function the action serves for the person; that is, whether the action enabled the person to act as a causal agent and enhances the development of self-determination [18].

*Volitional action* is based on conscious choices that reflect one’s preference. That is, volitional actions are self-initiated and function to enable a person to act autonomously and to engage in self-governed action. Volitional actions involve the initiation and activation of causal capabilities—the capacity to cause something to happen—in one’s life. Agentic actions refer to the means by which something is done or achieved; they are self-directed and goal-focused. When acting agentically, self-determined people identify pathways that lead to a specific end or cause or create change. The identification of pathways, or pathways thinking, is a proactive, purposive process. Agentic action is self-regulated, self-directed, and enables progress toward freely chosen goals. Volitional actions involve the initiation and activation of agentic capabilities—the capacity to sustain action toward a goal [18].

Finally, in acting volitionally and agentically, self-determined people develop a sense of personal empowerment and a belief that they have what it takes to achieve their freely chosen goals. They perceive linkages between their actions and the outcomes they want to experience; they develop adaptive action-control beliefs. To account for these beliefs and actions, Causal Agency Theory incorporates basic tenets of Action-Control Theory [19], which posits three types of action-control beliefs: beliefs about the link between the self and the goal (control expectancy beliefs; “When I want to do ____, I can”); beliefs about the link between the self and the means for achieving the goal (capacity beliefs; “I have the capabilities to do _____”); and beliefs about the utility or usefulness of a given means for attaining a goal (causality beliefs; “I believe my effort will lead to goal achievement” vs. “I believe other factors—luck, access to teachers, or social capital—will lead to goal achievement”). As adaptive action-control beliefs emerge, people are better able to act with self-awareness and self-knowledge in a goal-directed manner [19].

Through each iteration of this theoretical work and research in self-determination, the relationship between self-determination and quality of life has remained a critical feature. Self-determination, in Causal Agency Theory, is seen as contributing to one’s overall quality of life. In the following section, the role of self-determination within theories of quality of life is discussed, followed be a look at the research pertaining to quality of life and self-determination in the disability context.

## 4. Quality of Life and Self-Determination

Schalock and Verdugo [20] pointed out that the quality-of-life concept has provided an outcomes-based evaluation framework associated with specific domains (including self-determination) that enable both the consideration of personally valued life outcomes and the design of large systems of supports. However, we do not have to take the relationship between quality of life and self-determination as a theoretical supposition only. There has been some research examining the relationship among these two constructs or as part of the examination of relative contributions of all the core dimensions of quality of life. Schalock and colleagues [20] conducted a study examining the core dimensions of quality of life across five geographical groups (Spain, Central/South America, Canada, Mainland China, and the United States). The purpose of the study was to identify properties of these core dimensions across geographically and culturally diverse populations. More than 750 respondents completed a survey that asked questions about relative importance of each of eight core dimension (including self-determination) and 24 indicators of those dimensions, and the degree to which each indicator was available to the person/supported by the system. Respondents indicated importance and availability on a four-point scale, with 1 indicating not important or never and 4 indicating very important or always. Participants included people with intellectual and developmental disabilities, professionals in the field, and families of people with intellectual and developmental disabilities. Across type of respondents and geographical regions, all dimensions scored high (around 3 out of 4 up to 3.5 out of 4) on ratings of the importance of each of these. Interestingly, when reporting on the importance of the core dimension of self-determination, people with disabilities themselves rated it higher than did either professionals or family members. Based upon analyses of variance by group (person with disability, professional, parent) and geographic region (Spain, Central/South America, Canada, Mainland China, and the United States), there were significant differences in reports of importance of self-determination as a function of group (with, as previously noted, people with disability reporting the highest importance and professionals reporting the lowest) as well as geographic regions (with importance of self-determination generally lower (though still at or around 3 out of 4) in China and highest in Spain and North America.

Two studies have directly measured quality of life and self-determination to examine relationships among people with intellectual and developmental disabilities. Wehmeyer and Schwartz [21] measured the self-determination and quality of 50 adults with an intellectual disability. Quality of life was measured by using the Quality of Life Questionnaire [22], and self-determination status was measured by The Arc’s Self-Determination Scale [23]. The number of choices available to each participant was measured by using the Life Choices Survey [24]. These researchers hypothesized that individual self-determination status and choice opportunity would predict high- or low-quality-of-life group membership. Based on a discriminant function analysis, it was determined that self-determination scores predicted membership in the high-quality-of-life group, and that such scores correlated significantly with choice opportunities. Lachapelle and colleagues [25] replicated this study, though measuring only self-determination and quality of life, with a sample of people with intellectual and developmental disabilities in four countries: Canada, the United States, Belgium, and France. Those findings mirrored those of Wehmeyer and Schwartz, indicating that overall self-determination as well as subdomains of self-determined behavior (including autonomous functioning) predicted membership in the higher quality of life group.

Choice and choice opportunity have been linked with self-determination and quality of life. Research has established that the environments in which many people with an intellectual disability live or work—and particularly congregate settings—restrict opportunities for choice-making and the expression of preferences [26]. Wehmeyer and Metzler [27] conducted a secondary analysis of data from a nationwide US survey of more than 4500 people with intellectual disability, finding that participants had limited choice opportunities across virtually every aspect of their lives, from whom they lived with to where they ate or worked. Stancliffe and Wehmeyer [28] used the Life Choices Survey [24] with nearly 400 people with an intellectual disability, and they found that living arrangement impacted choice opportunity with more inclusive living arrangements supporting enhanced choice opportunities, but, that, in general, beyond mundane day-to-day tasks, people with intellectual disability were provided very few opportunities to make choices, particularly when it came to more meaningful choices such as where to work. Two studies examining the relationship between choice opportunity, self-determination, and living/working arrangements directly confirmed the link between choice and self-determination, as well as to lifestyle satisfaction. Wehmeyer and Bolding [29] conducted a matched-samples study of adults with intellectual disability who were matched by age and intelligence level but varied as to whether they lived or worked in a large congregate, community-based congregate, or community-based setting. Level of self-determination, choice opportunity, and lifestyle satisfaction varied by living or working setting, with people living or working in more typical community-based settings higher in all those areas. In a subsequent study, these researchers [30] measured the self-determination and choice opportunities of people with intellectual disability who were moving from a more restrictive (congregate) work or living situation to an integrated, community-based work or living situation. There were significant positive changes in self-determination and choice opportunity as a function of moving from the more restrictive to the community-based setting. Finally, there are several studies of people with intellectual and developmental disabilities who have moved from institutions into the community that document the connection between choice opportunity and quality of life. Kozma and colleagues [31] summarized this literature, finding that people who moved to the community had, among other factors, increased opportunities for choice and experienced better quality of life and life satisfaction.

## 5. Discussion

There is both an intuitive and theoretical link between self-determination and quality of life for people with intellectual and developmental disabilities. Intuitively, it makes sense that greater autonomy and volitional action would enhance one’s quality of life. Theoretically, definitions of self-determination have framed the construct with regard to its contribution to a person’s overall quality of life, while theoretical frameworks of quality of life have included self-determination as among the core dimensions contributing to enhanced quality of life. These theoretical linkages have been supported by research, discussed previously, on the quality of life and self-determination of people with intellectual and developmental disabilities and the relationship between those constructs. There are several implications from this knowledge base that warrant consideration. First, most of this research has been survey research or quasi-experimental, and there is a need for more rigorous examinations of the two constructs. Both constructs can only be measured by using self-report assessments, and thus such research is labor-intensive. Nevertheless, it would be important to have larger-scale studies that employ randomized trial designs to establish these relationships. Second, the field of positive psychology has expanded research in areas pertaining to well-being, happiness, optimism, and life satisfaction, all of which pertain to quality of life and with which self-determination interacts to improve life outcomes for people. For example, Shogren and colleagues [32] conducted a structural equation modeling analysis of the relationships among the psychological constructs of hope, optimism, locus of control, and self-determination in predicting life satisfaction of adolescents with and without cognitive disabilities. Hope and optimism predicted life satisfaction directly, but those effects were mediated (positively) by self-determination and locus of control. Thus, there is a need to examine the interrelatedness among and between self-determination, quality of life, and other closely associated constructs. Third, we know very little about the quality of life and self-determination of people with intellectual and developmental disabilities who cannot reliably complete self-report measures. There are some larger-scale, statewide surveys, such as the National Core Indicators project in the United States (https://www.nationalcoreindicators.org/), that use proxy reporting to provide indicators of quality of life, but there is a need for validated measures of self-determination and quality of life that do not rely on self-report. Finally, most of this research has been in the domains of education, independent living, or employment. There is a need to examine issues of self-determination and quality of life of people with an intellectual disability in the public-health domain. Issues of autonomy and volition are critically important in the public-health domain, and, obviously, greater opportunity to act as a causal agent in one’s health-related life should contribute to improved quality of life. However, that research has yet to be conducted and is needed.

## 6. Conclusions

It goes without saying that, individually, self-determination and quality of life are important constructs, particularly in designing supports that enable people with intellectual and developmental disabilities to live, learn, work, and play in their communities. Being self-determined—that is, acting volitionally and making things to happen in one’s life—has been linked to multiple positive outcomes, including, importantly, enhanced quality of life and life satisfaction. Therefore, one means to enhance the quality of life of people with intellectual and developmental disabilities is to promote and enable people to be self-determined.
